# Bone Marrow Involvement of Rhabdomyosarcoma from Unknown Origin

**DOI:** 10.4274/tjh.2013.0155

**Published:** 2014-06-10

**Authors:** Begüm Şirin Koç, Serap Karaman, Ayşegül Ünüvar, Ezgi Uysalol, Zeynep Karakaş, Ömer Devecioğlu, Sema Anak

**Affiliations:** 1 İstanbul University İstanbul Faculty of Medicine, Department of Pediatric Hematology&Oncology, İstanbul, Turkey

**Keywords:** Hematologic manifestations of systemic diseases, Lymphoid cells neoplasms, Bone marrow failure

## 2. MORPHOLOGY IN HEMATOLOGY

**Figure 1:**
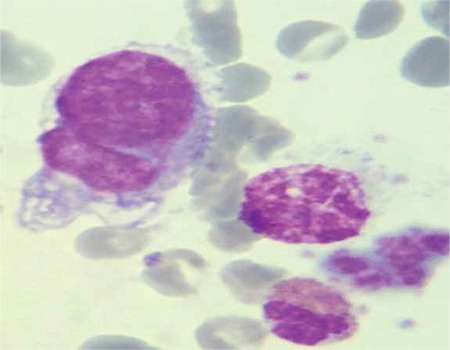
Bone marrow aspiration specimen from the patient with RMS.

**Figure 2:**
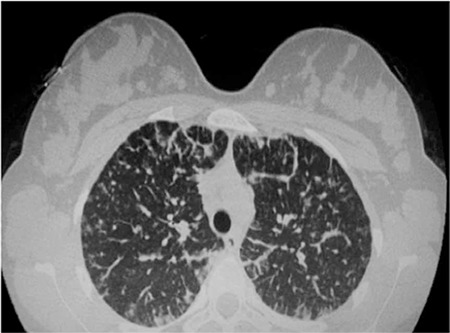
Lung and breast metastasis.

A 16-year-old girl was admitted to the hospital suffering from severe back pain, night sweats, and weight loss for 3 weeks. On physical examination, she had multiple lymphadenopathies in the cervical, axillary, and inguinal regions. There was no organomegaly. Laboratory examination revealed the following: Hb, 10.7 g/dL; Hct, 27%; leukocytes, 15.5x10^9^/L; neutrophils, 12.2x10^9^/L; platelets, 337x10^9^/L. Peripheral blood smear revealed no blasts or atypical cells. The erythrocyte sedimentation rate was 102 mm/h. All biochemical test results were within normal ranges, except for elevated lactate dehydrogenase enzyme and C-reactive protein. The bone marrow aspirate smear showed immature cells with vacuole and blue cytoplasm; these immature cells were considered as metastasis of a solid tumor (Figure 1). Excisional biopsy of the axillary lymphadenopathy was performed and the histopathological diagnosis was the alveolar type of rhabdomyosarcoma (RMS). Work-up of the primary site of the disease was performed with whole body imaging. Cranial, neck, abdominal, and pelvic MRI results were in normal range. Thorax CT showed metastatic nodules in the both lung; furthermore, multiple solid masses were detected in both breasts (Figure 2). The metastatic disease encompassed the bone marrow, thorax, and lymph nodes. Distant metastasis was present at diagnosis in this case but we could not find the primary site of the disease. We think that the bilateral breast masses may be the primary sites.

